# Modelling and Optimal Control of Typhoid Fever Disease with Cost-Effective Strategies

**DOI:** 10.1155/2017/2324518

**Published:** 2017-09-10

**Authors:** Getachew Teshome Tilahun, Oluwole Daniel Makinde, David Malonza

**Affiliations:** ^1^Pan African University Institute of Basic Sciences Technology and Innovation, Nairobi, Kenya; ^2^Faculty of Military Science, Stellenbosch University, Stellenbosch, South Africa; ^3^Department of Mathematics, Kenyatta University, Nairobi City, Kenya

## Abstract

We propose and analyze a compartmental nonlinear deterministic mathematical model for the typhoid fever outbreak and optimal control strategies in a community with varying population. The model is studied qualitatively using stability theory of differential equations and the basic reproductive number that represents the epidemic indicator is obtained from the largest eigenvalue of the next-generation matrix. Both local and global asymptotic stability conditions for disease-free and endemic equilibria are determined. The model exhibits a forward transcritical bifurcation and the sensitivity analysis is performed. The optimal control problem is designed by applying Pontryagin maximum principle with three control strategies, namely, the prevention strategy through sanitation, proper hygiene, and vaccination; the treatment strategy through application of appropriate medicine; and the screening of the carriers. The cost functional accounts for the cost involved in prevention, screening, and treatment together with the total number of the infected persons averted. Numerical results for the typhoid outbreak dynamics and its optimal control revealed that a combination of prevention and treatment is the best cost-effective strategy to eradicate the disease.

## 1. Introduction

**Figure 1:**
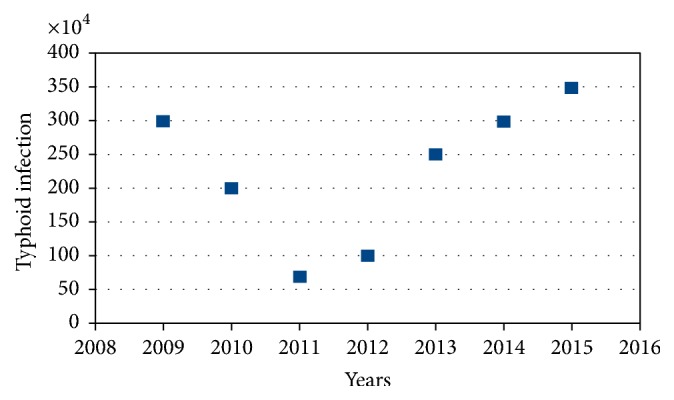
Reported cases of typhoid in Ethiopia for the past seven years.

According to [1], “infectious diseases are those diseases caused by viruses, bacteria, epiphytes, and parasites such as protozoans or worms that have a potential to spread into the population easily.” Typhoid fever is one of the common infectious diseases in human beings that is caused by different species of Salmonella. The most common species of Salmonella that cause typhoid fever are* Salmonella paratyphi A, B, and C* and* Salmonella paratyphi D* [WHO [2]]. “Most of the time typhoid fever is caused by lack of sanitation in which the disease causing bacteria is transmitted by ingestion of contaminated food or water” WHO, 2003. The bacteria are released from the infectious individuals or carriers and then contaminate food or drinking water as a consequence of unsatisfactory hygiene practices. Due to this, typhoid fever is a common disease in developing countries. The data taken from Ethiopia for that past seven years (2009–2015), in Figure 1, indicate that in each year the disease is increasing in alarming rate. Mathematical models have great benefits for describing the dynamics of infectious disease. Moreover, it plays a significant role in predicting suitable control strategies and analyzing and ranking their cost-effectiveness (for example, see Okosun and Makinde [3–7]). Very essential research results on the transmission dynamics of typhoid have come out in the last decade; for instance, see Adetunde [8], Mushayabasa and Bhunu [9], Moffat et al. (2014), Steady et al. (2014), Adeboye and Haruna [10], Omame et al. [11], Khan et al. [12], and Akinyi et al. [13]. All of the above studies reveal an important result for typhoid fever dynamics by considering different countries situation. But we have identified that till now there is no study that has been done to investigate the typhoid fever dynamics with the application of optimal control methods and cost-effectiveness analysis of the applied control strategies.

In view of the above, we developed a deterministic mathematical model to investigate the dynamics of typhoid fever with optimal control strategies and also we investigated the cost-effectiveness of the implemented control strategies.

## 2. Model Description and Formulation

The model considers human population as well as bacteria population (*B*_*c*_). The human population at time *t* is divided into four subclasses.* Susceptible* (*S*): this class includes those individuals who are at risk for developing an infection from typhoid fever disease.* Infected* (*I*): this class includes all individuals who are showing the symptom of the disease.* Carrier* (*C*): this is a person who is colonized by the bacterium* Salmonella typhi* without showing any obvious signs of disease and who is a potential source of infection to others by contaminating foods and water carelessly during preparation and handling.* Recovered* (*R*): this class includes all individuals that have recovered from the disease and got temporary immunity. The susceptible class is increased by birth or emigration at a rate of Λ and also from recovered class by losing temporary immunity with *δ* rate. Susceptible individuals will get typhoid causing bacteria when they take foods or waters which is contaminated by Salmonella bacteria. The force of infection of the model is *λ* = *B*_*c*_*v*/(*K* + *B*_*c*_), where *v* is ingestion rate, *K* is the concentration of Salmonella bacteria in foods or waters, and *B*_*c*_/(*K* + *B*_*c*_) is the probability of individuals in consuming foods or drinks contaminated with typhoid causing bacteria. After the susceptible individuals got the typhoid causing bacteria, they have probability of joining carrier with *ρ* rate or being a member of infective with 1 − *ρ* rate. The infected subclass is increased from carrier subclass by *θ* screening rate. Those individuals in the infected subclass can get treatment and join recovered subclass with a rate of *β*. The recovered subclass also increases with individuals who came from carrier class by getting natural immunity with a rate of *ϕ*. In all human subclasses, *μ* is the natural death rate of individuals, but in the infective class *α* is the disease causing death rate. The model assumed the bacteria population in contaminated foods and waters, where carriers and infectives can contribute to increasing the number of bacteria population in foods and waters without proper sanitation with a discharge rate of *σ*_1_ and *σ*_2_, respectively. We consider *μ*_*b*_ to be the death rate of Salmonella bacteria and all the described parameters are nonnegative.

**Figure 2:**
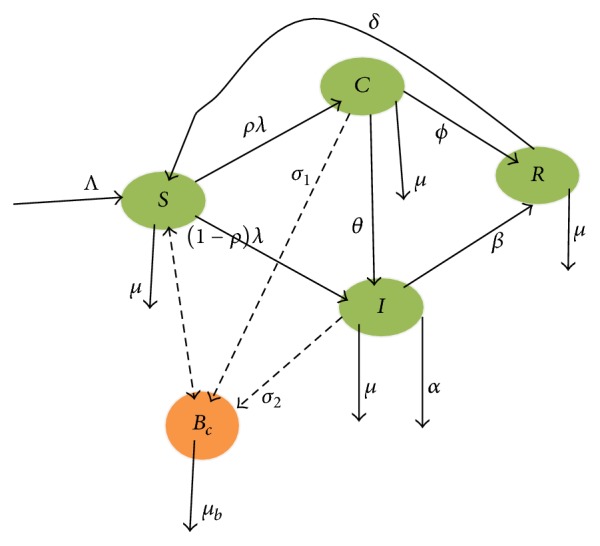
Flow diagram of the model.

The above model description is represented Figure 2.

Figure 2 can be written in five systems of differential equations.

(1) dSdt=Λ+δR−μ+λS,dCdt=ρλS−σ1+θ+μ+ϕC,dIdt=1−ρλS+θC−σ2+β+μ+αI,dRdt=βI+ϕC−μ+δR,dBcdt=σ1C+σ2I−μbBc,

where *λ* = *B*_*c*_*v*/(*K* + *B*_*c*_), with initial condition *S*(0) = *S*_0_, *C*(0) = *C*_0_, *I*(0) = *I*_0_, *R*(0) = *R*_0_, and *B*_*c*_(0) = *B*_*c*0_.

## 3. The Model Analysis

### 3.1. Invariant Region

We obtained the invariant region, in which the model solution is bounded. To do this, first we considered the total human population (*N*), where *N* = *S* + *C* + *I* + *R*.

Then, differentiating *N* both sides with respect to *t* leads to

(2) $$\begin{array}{c} \frac{dN}{dt}=\frac{dS}{dt}+\frac{dC}{dt}+\frac{dI}{dt}+\frac{dR}{dt}. \end{array}$$

By combining (1) and (2), we can get

(3) $$\begin{array}{c} \frac{dN}{dt}=\Lambda -\mu N-\alpha I. \end{array}$$

In the absence of mortality due to typhoid fever disease (*α* = 0), (3) becomes

(4) $$\begin{array}{c} \frac{dN}{dt}\leq \Lambda -\mu N. \end{array}$$

Integrating both sides of (4),

(5) ∫dNΛ−μN≤∫dt⟺−1μln⁡Λ−μN≤t+c

which simplifies into

(6) $$\begin{array}{c} \Lambda -\mu N\geq Ae^{-\mu t}, \end{array}$$

where *A* is constant. By applying the initial condition *N*(0) = *N*_0_ in (6), we get *A* = Λ − *μN*_0_ which upon substitution in (6) yields

(7) $$\begin{array}{c} \Lambda -\mu N\geq \left(\;\Lambda -\mu N_{0}\;\right)e^{-\mu t}. \end{array}$$

Then by rearranging (7), we can get

(8) $$\begin{array}{c} N\leq \frac{\Lambda }{\mu }-\left[\;\frac{\Lambda -\mu N_{0}}{\mu }\;\right]e^{-\mu t}. \end{array}$$

As *t* → *∞* in (8), the population size *N* → Λ/*μ* which implies that 0 ≤ *N* ≤ Λ/*μ*. Thus, the feasible solution set of the system equation of the model enters and remains in the region:

(9) $$\begin{array}{c} \Omega =\left\{\;\left(\;S,I,C,R\;\right)\in R_{+}^{4}:N\leq \frac{\Lambda }{\mu }\;\right\}. \end{array}$$

Therefore, the basic model is well posed epidemiologically and mathematically. Hence, it is sufficient to study the dynamics of the basic model in *Ω*.

### 3.2. Positivity of the Solutions

We assumed that the initial condition of the model is nonnegative, and now we also showed that the solution of the model is also positive.

**Theorem 1 .**

Let *Ω* = {(*S*, *C*, *I*, *R*, *B*_*c*_) ∈ *ℜ*_+_^5^ : *S*_0_ > 0, *I*_0_ > 0, *C*_0_ > 0, *R*_0_ > 0, *B*_*c*_0__ > 0}; then the solutions of {*S*, *C*, *I*, *R*, *B*_*c*_} are positive for *t* ≥ 0.

**Proof**

From the system of differential equation (1), let us take the first equation:

(10) dSdt=Λ+δR−μ+λS⟹dStdt≥−μ+λSt⟹dStSt≥−μ+λdt⟹∫dStSt≥−∫μ+λdt.

Then by solving using separation of variable and applying condition, we obtained

(11) $$\begin{array}{c} S\left(\;t\;\right)\geq S_{0}e^{-\left(\mu +\lambda \right)t}\geq 0. \end{array}$$

And also by taking the second equation of (1), that is,

(12) $$\begin{array}{c} \frac{dC}{dt}=\rho \lambda S-\left(\;\sigma_{1}+\theta +\mu +\phi \;\right)C, \end{array}$$

it is true that

(13) dCdt≥−σ1+θ+μ+ϕC⟹dCC≥−σ1+θ+μ+ϕdt⟹∫dCC≥−∫σ1+θ+μ+ϕdt.

Then by solving using separation of variable and applying initial condition gives;

(14) $$\begin{array}{c} ∴C\left(\;t\;\right)\geq C_{0}e^{-\left(\mu +\phi \right)t}\geq 0. \end{array}$$

Similarly we took the third equation of (1) which is;

(15) $$\begin{array}{c} \frac{dI\left(t\right)}{dt}=\left(\;1-\rho \;\right)\lambda S+\theta C-\left(\;\sigma_{2}+\beta +\mu +\alpha \;\right)I \end{array}$$

it is true that

(16) dIdt≥−σ2+β+μ+αI⟹dII≥−σ2+β+μ+αdt⟹∫dII≥−∫σ2+β+μ+αdt.

After solving using technique of separation of variable and then applying initial condition, the following is obtained:

(17) $$\begin{array}{c} ∴I\left(\;t\;\right)\geq I_{0}e^{-\left(\sigma_{2}+\beta +\mu +\alpha \right)t}\geq 0. \end{array}$$

We took the fourth equation of (1) which is

(18) dRdt=βI+ϕC−μ+δR⟹dRdt≥−μ+δR⟹dRR≥−μ+δdt⟹∫dRRt≥−∫μ+δdt∴Rt≥R0e−μ+δt≥0.

Finally we took the fifth equation of (1),

(19) dBcdt=σ1C+σ2I−μbBc⟹dBcdt≥−μbBc⟹dBcBct≥−μbdt⟹∫dBcBc≥−∫μbdt∴Bc≥Bc0e−μbt≥0.

This completes the proof of the theorem.

Therefore, the solution of the model is positive.

### 3.3. The Disease-Free Equilibrium (DFE)

To find the disease-free equilibrium (DFE), we equated the right hand side of model (1) to zero, evaluating it at *C* = *I* = 0 and solving for the noninfected and noncarrier state variables. Therefore, the disease-free equilibrium *E*_0_ = (Λ/*μ*, 0,0, 0,0).

### 3.4. The Basic Reproductive Number (*ℜ*_0_)

In this section, we obtained the threshold parameter that governs the spread of a disease which is called the basic reproduction number which is determined. To obtain the basic reproduction number, we used the next-generation matrix method so that it is the spectral radius of the next-generation matrix [15].

The model equations are rewritten starting with newly infective classes:

(20) dCdt=ρλS−σ1+θ+μ+ϕC,dIdt=1−ρλS+θC−σ2+β+μ+αI,dBcdt=σ1C+σ2I−μbBc.

Then by the principle of next-generation matrix, we obtained

(21) f=ρBcvK+BcS1−ρBcvK+BcS,v=σ1+θ+μ+ϕCσ2+β+μ+αI−θC−σ1C+σ2I−μbBc.

The Jacobian matrices of *f* and *v* evaluated at DFE are given by *F* and *V*, respectively, such that

(22) F=00ρΛvμK001−ρΛvμK000,V=σ1+θ+μ+ϕ00−θσ2+β+μ+α0−δ1−δ2μb.

The inverse of *V* is obtained and given by

(23) $$\begin{array}{c} V^{-1}=\left[\begin{array}{ccc} \frac{1}{k_{1}} & 0 & 0\\ \frac{\theta }{k_{1}k_{2}} & \frac{1}{k_{2}} & 0\\ \frac{\theta \sigma_{2}+\sigma_{1}k_{2}}{k_{1}k_{2}\mu_{b}} & \frac{\sigma_{2}}{k_{2}\mu_{b}} & \frac{1}{\mu_{b}} \end{array}\right], \end{array}$$

where *k*_1_ = (*σ*_1_ + *θ* + *μ* + *ϕ*) and *k*_2_ = (*σ*_2_ + *β* + *μ* + *α*).

Then,

(24) FV−1=ρΛvθσ2+σ1k2μKk1k2μbρΛvσ2μKk2μbρΛvvKμb1−ρΛvθσ2+σ1k2μKk1k2μb1−ρΛvσ2μKk2μb1−ρΛvvKμb000.

The characteristic equation of *FV*^−1^ is obtained as

(25) $$\begin{array}{c} \lambda^{2}\left(\;\rho \frac{\Lambda v\left(\theta \sigma_{2}+\sigma_{1}k_{2}\right)}{\mu Kk_{1}k_{2}\mu_{b}}+\left(\;1-\rho \;\right)\;\right)\frac{\Lambda v\sigma_{2}}{\mu Kk_{2}\mu_{b}}=0. \end{array}$$

The eigenvalues of *FV*^−1^ are

(26) λ1=λ2=0,λ3=ρΛvθσ2+σ1k2μKk1k2μb+1−ρΛvσ2μKk2μb.

The dominant eigenvalue of *FV*^−1^ is *λ*_3_.

Therefore, the basic reproduction number (*ℜ*_0_) after substituting *k*_1_ and *k*_2_ is given by

(27) R0ρθσ2+σ1σ2+β+μ+ασ1+θ+μ+ϕ+1−ρσ2·ΛvμKσ2+β+μ+αμb.

### 3.5. Local Stability of Disease-Free Equilibrium

**Proposition 2 .**

The disease-free equilibrium point is locally asymptotically stable if *ℜ*_0_ < 1 and unstable if *ℜ*_0_ > 1.

**Proof**

To proof this theorem first we obtain the Jacobian matrix of system (1) at the disease-free equilibrium *E*_0_ as follows:

(28) $$\begin{array}{c} J_{E_{0}}=\left[\begin{array}{ccccc} -\mu & 0 & 0 & \delta & \frac{v\Lambda }{K\mu }\\ 0 & -\left(\sigma_{1}+\theta +\mu +\phi \right) & 0 & 0 & \frac{\rho v\Lambda }{\mu K}\\ 0 & \theta & -\left(\sigma_{2}+\beta +\mu +\alpha \right) & 0 & \frac{\left(1-\rho \right)v\Lambda }{\mu K}\\ 0 & \phi & \beta & -\left(\mu +\delta \right) & 0\\ 0 & \sigma_{1} & \sigma_{2} & 0 & -\mu_{b} \end{array}\right]. \end{array}$$

From the Jacobian matrix of (28), we obtained a characteristic polynomial:

(29) −λ−μ−λ−μ+δλ3+L1λ2+L2λ+L3=0,

where

(30) L1=σ2+β+2μ+α+σ1+ϕ+θ+μb,L2=μbσ2+β+2μ+α+σ1+ϕ+θ+σ2+β+μ+ασ1+μ+ϕ+θ−ρσ1+1−ρσ2vΛμK,L3=μbσ2+β+μ+ασ1+μ+ϕ+θ1−R0.

From (29) clearly, we see that

(31) −λ−μ=0,or  −λ−μ+δ=0,or  λ3+L1λ2+L2λ+L3=0⇓λ1=−μ<0,λ2=−μ+δ<0.

For the last expression, that is,

(32) $$\begin{array}{c} \lambda^{3}+L_{1}\lambda^{2}+L_{2}\lambda +L_{3}=0, \end{array}$$

we applied Routh-Hurwitz criteria. By the principle of Routh-Hurwitz criteria, (32) has strictly negative real root if and only if *L*_1_ > 0, *L*_3_ > 0, and *L*_1_*L*_2_ > *L*_3_.

Obviously we see that *L*_1_ is positive because it is a sum of positive variables, but *L*_3_ to be positive 1 − *ℜ*_0_ must be positive, which leads to *ℜ*_0_ < 1. Therefore, DFE will be locally asymptotically stable if and only if *ℜ*_0_ < 1.

### 3.6. Global Stability of DFE

**Theorem 3 .**

The disease-free equilibrium is globally asymptotically stable in the feasible region *Ω* if *ℜ*_0_ < 1.

**Proof**

To proof this theorem, we first developed a Lyapunov function, technically.

(33) $$\begin{array}{c} L=\left[\;\frac{\theta \sigma_{2}+\sigma_{1}k_{2}}{k_{1}}\;\right]C+\sigma_{2}I+k_{2}B_{c}, \end{array}$$

where *k*_1_ = *σ*_1_ + *θ* + *μ* + *ϕ* and *k*_2_ = *σ*_2_ + *β* + *μ* + *α*

Then differentiating *L* both sides leads to

(34) $$\begin{array}{c} \frac{dL}{dt}=\left[\;\frac{\theta \sigma_{2}+\sigma_{1}k_{2}}{k_{1}}\;\right]\frac{dC}{dt}+\sigma_{2}\frac{dI}{dt}+k_{2}\frac{dB_{c}}{dt}. \end{array}$$

Substituting expression for *dC*/*dt*, *dI*/*dt*, and *dB*_*c*_/*dt* from (1) to (34) results in

(35) dLdt=θσ2+σ1k2k1ρλS−σ1+θ+μ+ϕC+σ21−ρλS+θC−σ2+β+μ+αI+k2σ1C+σ2I−μbBc.

By collecting like terms of (35),

(36) dLdt=ρθσ2+σ1k2k1+1−ρσ2λS+θσ2−θσ2−σ1k2C−σ2k2I+k2σ1C+σ2I−μbBc.

Equation (36) can be simplified as

(37) $$\begin{array}{c} \frac{dL}{dt}=\left[\;\rho \frac{\theta \sigma_{2}+\sigma_{1}k_{2}}{k_{1}}+\left(\;1-\rho \;\right)\sigma_{2}\;\right]\lambda S-k_{2}\mu_{b}B_{c}\text{)}. \end{array}$$

Equation (37) can be written as interims of *ℜ*_0_,

(38) $$\begin{array}{c} \frac{dL}{dt}=\left(\;R_{0}\mu_{b}k_{2}\frac{\mu K}{\Lambda v}\;\right)\lambda S-k_{2}\mu_{b}B_{c}\text{)}. \end{array}$$

At *S* = *S*_0_ = Λ/*μ*, (38) becomes

(39) $$\begin{array}{c} \frac{dL}{dt}\leq \left(\;R_{0}-1\;\right)k_{2}\mu_{b}B_{c}. \end{array}$$

So *dL*/*dt* ≤ 0 if *ℜ*_0_ ≤ 1. Furthermore, *dL*/*dt* = 0⇔*B*_*c*_ = 0 which leads to *C* = *I* = 0 or *ℜ*_0_ = 1.

Hence, *L* is Lyapunov function on *Ω* and the largest compact invariant set in {(*S*, *C*, *I*, *R*, *B*_*c*_) ∈ *Ω*, *dL*/*dt* = 0} is the singleton (*S*_0_, 0,0, 0,0).

Therefore, by LaSalle's invariance principle (LaSalle [16]), every solution to equations of model (1) with initial conditions in *Ω* which approaches the disease-free equilibrium at *t* (time) tends to infinity (*t* → *∞*) whenever *ℜ*_0_ ≤ 1. Hence, the disease-free equilibrium is globally asymptotically stable.

### 3.7. The Endemic Equilibrium

The endemic equilibrium is denoted by *E*^*∗*^ = (*S*^*∗*^, *C*^*∗*^, *I*^*∗*^, *R*^*∗*^, *B*_*c*_^*∗*^) and it occurs when the disease persists in the community. To obtain it, we equate all the model equations (1) to zero. Then we obtain

(40) S∗=Λσ2+μ+α+βσ1+μ+θ+ϕμ+δμ+λ∗−βλ∗δ1−ρσ1+μ+θ+ϕ+ρθ−δϕρλ∗σ2+μ+β+α,C∗=ρλ∗Λσ2+μ+α+βμ+δμ+λ∗−βλ∗δ1−ρσ1+μ+θ+ϕ+ρθ−δϕρλ∗σ2+μ+β+α,I∗=R0Kσ1+μ+θ+ϕσ2+μ+β+αμμb−σ1ρΛvσ2+μ+β+αμ+δμKσ2+vσ2−βδR0Kσ1+μ+θ+ϕσ2+μ+β+αμμb+βδσ1σ2+μ+β+αΛv−δσ2ϕρvσ2+μ+β+α,R∗=βI∗+ϕC∗μ+δ,Bc∗=λ∗Λμ+λ∗σ1ρσ2+μ+α+β+σ21−ρσ1+μ+θ+ϕ+ρθμbμ+λ∗)−βλδ1−ρσ1+μ+θ+ϕ+ρθ−δϕρλ∗σ2+μ+β+α.

When we substitute the expression for *B*_*c*_^*∗*^ into the force of infection, that is, *λ*^*∗*^ = *B*_*c*_^*∗*^*v*/(*K* + *B*_*c*_^*∗*^), we obtained a characteristic polynomial of force of infection,

(41) $$\begin{array}{c} p\left(\;\lambda^{*}\;\right)=D_{1}{\lambda^{*}}^{2}+D_{2}\lambda^{*}=0, \end{array}$$

where *D*_1_ = 1 + *ℜ*_0_(*σ*_2_ + *μ* + *α* + *β*)(*σ*_1_ + *μ* + *θ* + *ϕ*)(*μ* + *δ*)*μμ*_*b*_*K* + (*βδ*((1 − *ρ*)(*σ*_1_ + *μ* + *θ* + *ϕ*) + *ρθ*) + *δϕρ*(*σ*_2_ + *μ* + *α* + *β*)), *D*_2_ = (1 − *ℜ*_0_)(*μ* + *δ*)*μ*.

Clearly, *D*_1_ > 0 and *D*_2_ ≥ 0. Whenever *ℜ*_0_ < 1, *λ*^*∗*^ = −*D*_1_/*D*_2_ ≤ 0. From this, we see that, for *ℜ*_0_ < 1, there is no endemic equilibrium for this model.

**Figure 3:**
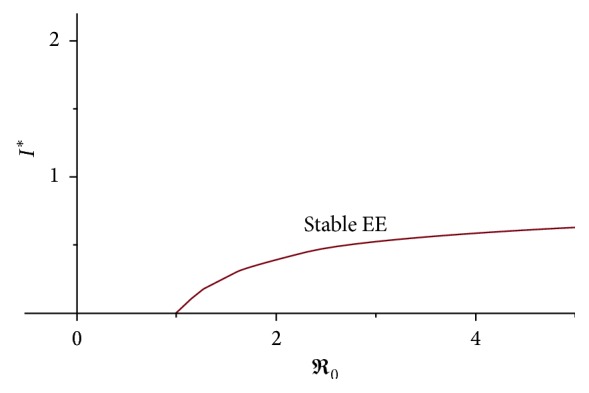
Forward bifurcation of typhoid fever model.

Therefore, this condition shows that it is not possible for backward bifurcation in the model if *ℜ*_0_ < 1. When we plot *I*^*∗*^ over *ℜ*_0_ by using the expression for *I*^*∗*^ and estimated parameters in Table 2, we got a forward bifurcation (Figure 3).

**Lemma 4 .**

A unique endemic equilibrium point *E*^*∗*^ exists and is positive if *ℜ*_0_ > 1.

## 4. Sensitivity Analysis of Model Parameters

On the basic parameters, we carried out sensitivity analysis. This helped us to check and identify parameters that can impact the basic reproductive number. To carry out sensitivity analysis, we followed the technique outlined by [17, 18]. This technique develops a formula to obtain the sensitivity index of all the basic parameters, defined as Δ_*x*_^*ℜ*_0_^ = (∂*ℜ*_0_/∂*x*)(*x*/*ℜ*_0_), for *x* represents all the basic parameters.

For example, the sensitivity index of *ℜ*_0_ with respect to *v* is Δ_*v*_^*ℜ*_0_^ = (∂*ℜ*_0_/∂*v*)(*v*/*R*_eff_) = 1. And with respect to the remaining parameters, Δ_*K*_^*ℜ*_0_^, Δ_*σ*_1__^*ℜ*_0_^, Δ_*σ*_2__^*ℜ*_0_^, Δ_*ρ*_^*ℜ*_0_^, Δ_*μ*_^*ℜ*_0_^, Δ_*μ*_*b*__^*ℜ*_0_^, Δ_*α*_^*ℜ*_0_^, Δ_*θ*_^*ℜ*_0_^, Δ_*β*_^*ℜ*_0_^, and Δ_*ϕ*_^*ℜ*_0_^ are obtained and evaluated at Λ = 100, *ϕ* = 0.0003, *σ*_1_ = 0.9, *σ*_2_ = 0.8, *β* = 0.0002, *ρ* = 0.3, *μ* = 0.0247, *μ*_*b*_ = 0.0001, *α* = 0.052, *θ* = 0.2, *v* = 0.9, and *K* = 50,000. Their sensitivity indices are in Table 1.

### 4.1. Interpretation of Sensitivity Indices

The sensitivity indices of the basic reproductive number with respect to main parameters are arranged orderly in Table 1. Those parameters that have positive indices (*v*, *K*, *σ*_1_, *σ*_2_, and *ρ*) show that they have great impact on expanding the disease in the community if their values are increasing. Due to the reason that the basic reproduction number increases as their values increase, it means that the average number of secondary cases of infection increases in the community. And also those parameters in which their sensitivity indices are negative (*μ*, *μ*_*b*_, *α*, *θ*, *β*, and *ϕ*) have an influence of minimizing the burden of the disease in the community as their values increase while the others are left constant. And also as their values increase, the basic reproduction number decreases, which leads to minimizing the endemicity of the disease in the community.

## 5. Extension of the Model into an Optimal Control

In this section, the basic model of typhoid fever is generalized by incorporating three control interventions. The controls are prevention (*u*_1_) (sanitation and proper hygiene controls), treatment (*u*_2_) (treating individuals who developed symptoms of the disease), and screening of carriers (*u*_3_) which helps them to get proper treatment if they are aware of their status.

After incorporating the controls into the basic model of typhoid fever, we get the following state equations:

(42) dSdt=Λ+δR−1−u1λS−μS,dCdt=1−u1ρλS−θ+u3C−σ1+ϕ+μC,dIdt=1−u11−ρλS+1−u3θC−u2+βI−σ2+μ+αI,dRdt=u2+βI+ϕC−μ+δR,dBcdt=σ1C+σ2I−μbBc,

where *λ* = *B*_*c*_*v*/(*K* + *B*_*c*_).

{0 ≤ *u*_1_ < 1, 0 ≤ *u*_2_ < 1, 0 ≤ *u*_3_ < 1, 0 ≤ *t* ≤ *T*} is Lebesgue measurable. Our main objective is to obtain the optimal levels of the controls and associated state variables that optimize the objective function. The form of the objective function is taken from [19] and given by

(43) $$\begin{array}{c} J=\underset{u_{1},u_{2},u_{3}}{\min}\overset{t_{f}}{\underset{0}{\int }}\left(\;b_{1}C+b_{2}I+\frac{1}{2}\overset{3}{\underset{i=1}{\sum }}w_{i}u_{i}^{2}\;\right)dt. \end{array}$$

The coefficients associated with state variables (*b*_1_ and *b*_2_) and with controls (*w*_*i*_) are positive. Due to the fact that cost is not linear in its condition, we make the cost expression ((1/2)*w*_*i*_*u*_*i*_^2^) quadratic.

As objective function (43) shows, we aimed to minimize the number of carriers, infectives, and costs. That is, we want to get an optimal triple (*u*_1_^*∗*^, *u*_2_^*∗*^, *u*_3_^*∗*^) such that

 
*J*(*u*_1_^*∗*^, *u*_2_^*∗*^, *u*_3_^*∗*^) = min{*J*(*u*_1_, *u*_2_, *u*_3_)∣*u*_*i*_ ∈ *U*}, where *U* = {(*u*_1_, *u*_2_, *u*_3_)∣each  *u*_*i*_ is measurable with 0 ≤ *u*_*i*_ < 1 for 0 ≤ *t* ≤ *t*_*f*_} is the set of acceptable controls.

### 5.1. Existence of an Optimal Control

The existence of the optimal control can be showed by using an approach of [20]. We have already justified the boundedness of the solution of the basic typhoid fever model. This result can be used to prove the existence of optimal control. For detailed proof, see [20] [Theorem  4.1, p68-69].

### 5.2. The Hamiltonian and Optimality System

To obtain the Hamiltonian (*H*), we follow the approach of [21] such that

(44) $$\begin{array}{c} H=\frac{dJ}{dt}+\lambda_{1}\frac{dS}{dt}+\lambda_{2}\frac{dC}{dt}+\lambda_{3}\frac{dI}{dt}+\lambda_{4}\frac{dR}{dt}+\lambda_{5}\frac{dB_{c}}{dt}. \end{array}$$

That is,

(45) HS,C,I,R,Bc,t=LC,I,u1,u2,u3,t+λ1dSdt+λ2dCdt+λ3dIdt+λ4dRdt+λ5dBcdt,

where *L*(*C*, *I*, *u*_1_, *u*_2_, *u*_3_, *t*) = *b*_1_*C* + *b*_2_*I* + (1/2)∑_*i*=1_^3^*w*_*i*_*u*_*i*_^2^, *λ*_1_, *λ*_2_, *λ*_3_, *λ*_4_, and *λ*_5_ are the adjoint variable functions. To obtain the adjoint variables, we followed the classical result of [21].

**Theorem 5 .**

There exist an optimal control set of *u*_1_, *u*_2_, and *u*_3_ and corresponding solution, *S*, *C*, *I*, *R*, and *B*_*c*_, that minimize *J*(*u*_1_, *u*_2_, *u*_3_) over *U*. Furthermore, there exist adjoint functions *λ*_1_,…, *λ*_5_ such that

(46) dλ1dt=−λ1−μ−Bcv1−u1K+Bc−λ21−ρ1−u1BcvK+Bc−λ31−u1ρvBcK+Bc,dλ2dt=−b1−λ2−θ−u3−λ31−u3θ−λ4ϕ−λ5σ1+ϕ+μ,dλ3dt=−b2−λ3−u2−β−σ2−λ4u2+β−λ5σ2+μ+α,dλ4dt=−λ1δ−λ4−μ−δ,dλ5dt=−λ1Bcv1−u1sk+Bc2−λ21−u1ρvSK+Bc−1−u1ρvB−cSK+Bc2−λ31−ρ1−u1vSK+Bc−1−ρ1−u1BcvSK+Bc2+λ5μb,

with transversality conditions,

(47) $$\begin{array}{c} \lambda_{i}\left(\;t_{f}\;\right)=0,\;i=1,\ldots ,5. \end{array}$$

And the characterized control set of (*u*_1_^*∗*^, *u*_2_^*∗*^, *u*_3_^*∗*^) is

(48) u1∗t=max⁡0,min⁡1,Sλ2ρvBc−Bcρvλ3+Bcvλ3−λ1BcvK+Bcw1,u2∗t=max⁡0,min⁡1,Iλ3−λ4w2,u3∗t=max⁡0,min⁡1,Cλ3θ+λ2w3.

**Proof**

To prove this theorem, we used the classical result of [21]. Accordingly, to get the system of adjoint variables, we differentiate the Hamiltonian (45) with respect to each state as follows:

(49) dλ1dt=−dHdS=−λ1−μ−Bcv1−u1K+Bc−λ21−ρ1−u1BcvK+Bc−λ31−u1ρvBcK+Bc,dλ2dt=−dHdC=−b1−λ2−θ−u3−λ31−u3θ−λ4ϕ−λ5σ1+ϕ+μ,dλ3dt=−dHdI=−b2−λ3−u2−β−σ2−λ4u2+β−λ5σ2+μ+α,dλ4dt=−dHdR=−λ1δ−λ4−μ−δ,dλ5dt=−dHdBc=−λ1Bcv1−u1SK+Bc2−λ21−u1ρvSK+Bc−1−u1ρvBcSK+Bc2−λ31−ρ1−u1vSK+Bc−1−ρ1−u1BcvSK+Bc2+λ5μb.

And also for characterization of the optimal control, we used the following partial differential equation:

(50) $$\begin{array}{c} \frac{\partial H}{\partial u_{i}}=0\;\text{at }u_{i}=u_{i}^{*}, \end{array}$$

where *i* = 1,2, 3.

For *i* = 1,

(51) ∂H∂u1=0at  u1∗⇓u1∗=Sλ2ρvBc−Bcρvλ3+Bcvλ3−λ1BcvK+Bcw1.

For *i* = 2,

(52) ∂H∂u2=0at  u2∗⇓u2∗=Iλ3−λ4w2.

For *i* = 3,

(53) ∂H∂u3=0at  u3∗⇓u3∗=Cλ3θ+λ2w3.

Since 0 < *u*_*i*_^*∗*^ < 1, we can write in a compact notation:

(54) u1∗=max⁡0,min⁡1,Sλ2ρvBc−Bcρvλ3+Bcvλ3−λ1BcvK+Bcw1,u2∗=max⁡0,min⁡1,Iλ3−λ4w2,u3∗=max⁡0,min⁡1,Cλ3θ+λ2w3.

### 5.3. The Optimality System

It is a system of states (42) and adjoint (46) incorporating with the characterization of the optimal control and initial and transversality conditions. Then we have the following optimality system:

(55) dSdt=Λ+δR−1−u1∗λS−μS,dCdt=1−u1∗ρλS−θ+u3∗C−σ1+ϕ+μC,dIdt=1−u1∗1−ρλS+1−u3∗θC−u2∗+βI−σ2+μ+αI,dRdt=u2∗+βI+ϕC−μ+δR,dBcdt=Q+σ1C+σ2I−μbBc,dλ1dt=−λ1−μ−Bcv1−u1∗k+Bc−λ21−ρ1−u1∗BcvK+Bc−λ31−u1∗ρvBcK+Bc,dλ2dt=−b1−λ2−θ−u3∗−λ31−u3∗θ−λ4ϕ−λ5σ1,dλ3dt=−b2−λ3−u2∗−β−σ2+μ+α−λ4u2∗+β−λ5σ2,dλ4dt=−λ1δ−λ4−μ−δ,dλ5dt=−λ1Bcv1−u1∗SK+Bc2−λ21−u1∗ρvSK+Bc−1−u1∗ρvB−cSK+Bc2−λ31−ρ1−u1∗vSK+Bc−1−ρ1−u1∗BcvSK+Bc2+λ5μb,λitf=0,i=1,2,3,4,5,S0=S0,C0=C0,I0=I0,R0=R0,Bc0=Bc0.

### 5.4. Uniqueness of the Optimality System

Since the state and adjoint variables are bounded and also the obtained ordinary differential equations have Lipschitz in their structure, it is possible to show the uniqueness, hence the following theorem.

**Theorem 6 .**

For *t* ∈ [0, *t*_*f*_], the bounded solutions to the optimality system are unique.

**Proof**

See [22] for the proof of this theorem.

## 6. Numerical Simulations

We perform numerical simulation of the optimality system by using the parameter values given in Table 2.

To obtain optimal solution, we apply iterative technique. By using an advantage of the initial conditions of the state system, we used a forward fourth-order Runge-Kutta method to solve it and also due to the final conditions for the adjoint system, we used a backward fourth-order Runge-Kutta method to solve it. To solve the state initial guess of controls is used and the solution of the state system and the initial guess helps to solve the adjoint system. Each control continues to be updated by combining its previous and characterization values. To repeat the solutions, the updated controls are used. This situation continues until two consecutive iterations are close enough [23].

To examine the impact of each control on eradication of typhoid fever disease, we used the following strategy:

- Applying prevention only (*u*_1_) as intervention
- Applying treatment only (*u*_2_) as intervention
- Applying screening only (*u*_3_) as intervention
- Implementing prevention (*u*_1_) and treatment (*u*_2_) intervention
- Implementing prevention (*u*_1_) and screening (*u*_3_) intervention
- Implementing treatment (*u*_2_) and screening (*u*_3_) intervention
- Using all the three controls: prevention effort *u*_1_, treatment effort *u*_2_, and also screening *u*_3_

Initial values that we used for simulation of the optimal control are *S*(0) = 1000, *C*(0) = 150, *I*(0) = 200, *R*(0) = 300, and *B*_*c*_(0) = 200 and also coefficients of the state and controls that we used are *b*_1_ = 25, *b*_2_ = 25, *w*_1_ = 4, *w*_2_ = 3, and *w*_3_ = 5.

### 6.1. Control with Prevention Only

**Figure 4:**
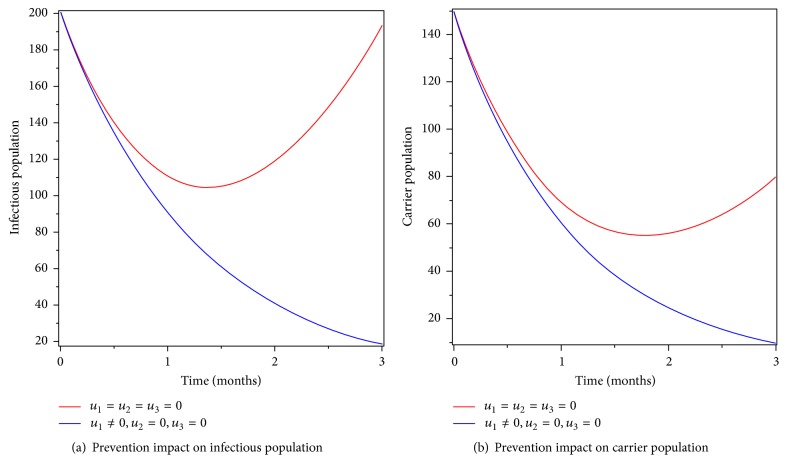
Simulations of typhoid fever model with prevention control only.

We simulated the optimality system by incorporating prevention intervention only. Figures 4(a) and 4(b) show the decrease of infectious and carrier population in the specified time. We conclude that prevention that includes sanitation and other techniques is a vital method to reduce typhoid fever infection. The number of individuals who have been with typhoid fever disease before implementation of prevention control has gone down due to disease induced and natural deaths. Therefore, applying optimized prevention control can eradicate typhoid fever disease in the community.

### 6.2. Control with Treatment Only

**Figure 5:**
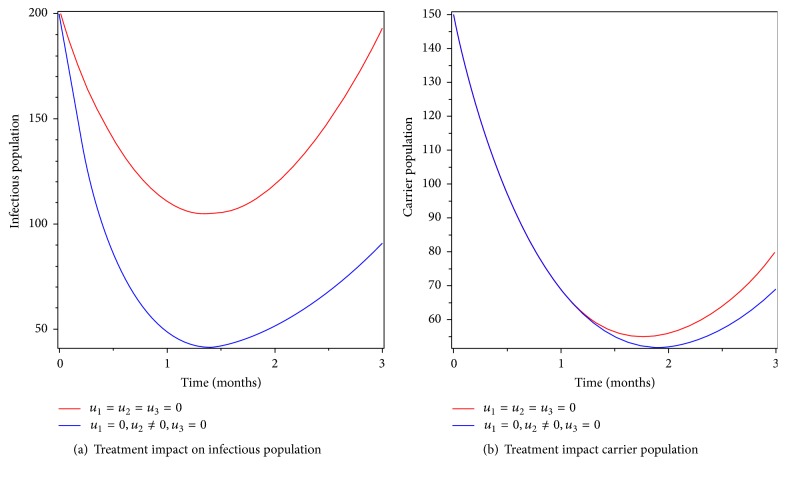
Simulations of typhoid fever model with treatment control only.

We applied treatment only as intervention that is treating individuals who develop disease symptom. From Figures 5(a) and 5(b), we understand that the number of infectious individuals and carriers decreased when treatment intervention is applied. The number of infectious individuals and carriers did not go to zero over the period of implementation of this intervention strategy. The reason is that due to lack of prevention susceptible individuals still get infected. Therefore, we conclude that applying optimized treatment only as control intervention decreases the burden of the disease but it cannot eradicate typhoid fever disease in the community.

### 6.3. Control with Screening Only

**Figure 6:**
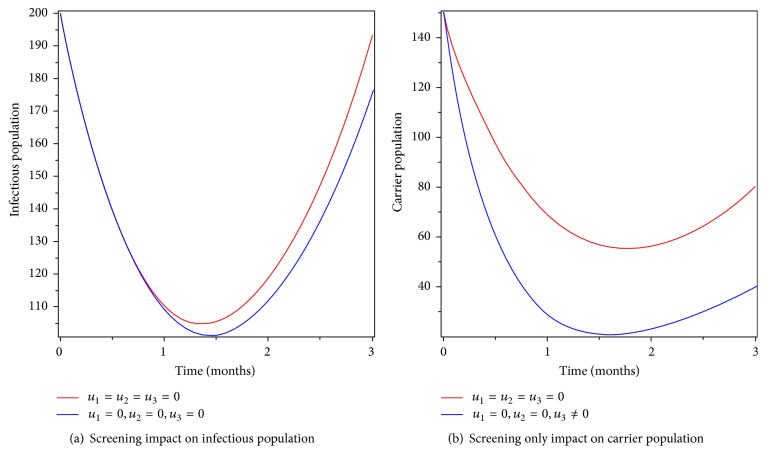
Simulations of typhoid fever model with screening control only.

As we know screening helps carriers to identify their status as they are leaving with the bacteria or not. Therefore, Figures 6(a) and 6(b) show that the infectious and carrier population goes down by screening effort but their number cannot be zero. New infection always appears in the community because the diseases are not prevented and individuals who develop the symptom of the disease are not getting treatment. Therefore, control with screening only reduces the burden in some extent but it is not helpful to eradicate typhoid fever disease totally from the community.

### 6.4. Control with Prevention and Treatment

**Figure 7:**
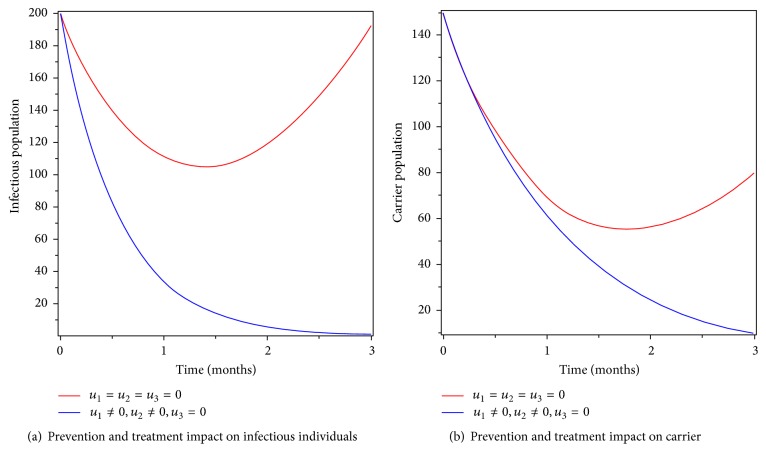
Simulations of typhoid fever model with prevention and treatment controls.

We simulate the model using a combination of prevention and treatment as intervention strategy for control of typhoid fever disease in the community. Figures 7(a) and 7(b) clearly show that the infectious and carrier population has gone to zero at the end of the implementation period. Therefore, we conclude that this strategy is effective in eradicating the disease from the community in a specified period of time.

### 6.5. Control with Prevention and Screening

**Figure 8:**
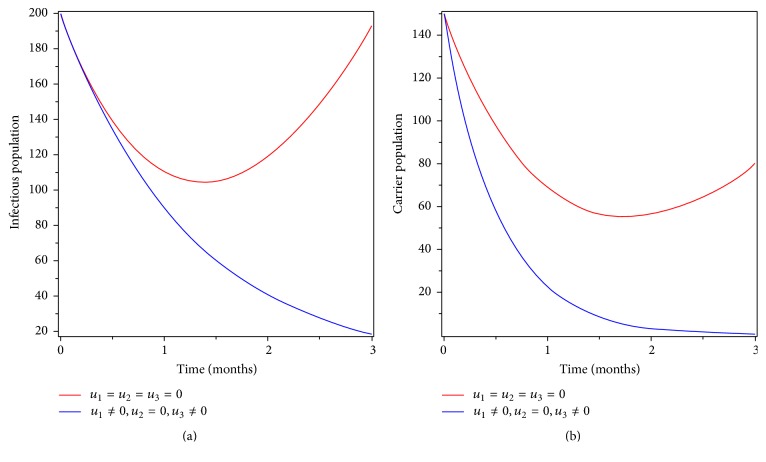
Simulations of the typhoid fever model with prevention and screening controls.

We simulated the model by incorporating optimized prevention and screening as disease control strategy. Figures 8(a) and 8(b) show that the infectious and carrier population goes to zero at the end of the implementation of intervention time. From this, we can conclude that applying prevention and screening can eradicate the disease even if without treating individuals that have disease symptom. Therefore, applying optimized prevention and screening as intervention strategy will eradicate typhoid fever disease from the community.

### 6.6. Control with Treatment and Screening

**Figure 9:**
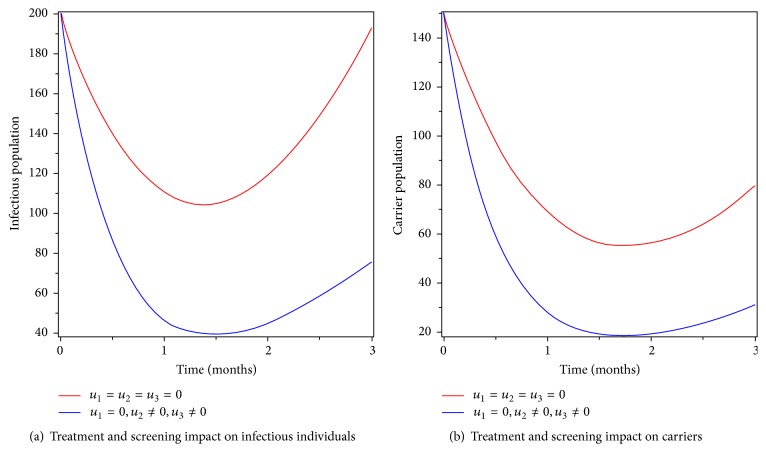
Simulations of the typhoid fever model with treatment and screening controls.

In this strategy, we applied treatment and screening as intervention to control typhoid fever disease. Figures 9(a) and 9(b) show that optimized intervention by treating infectious individuals and screening of carriers decreases the number of infectious and carrier populations but did not go to zero. Therefore, this strategy is not 100% effective in eradicating the disease in the specified period of time.

### 6.7. Control with Prevention, Treatment, and Screening

**Figure 10:**
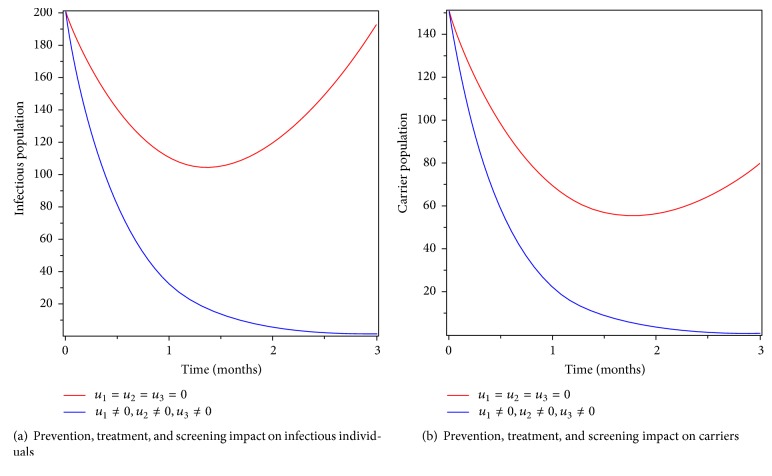
Simulations of the typhoid fever model with prevention, treatment, and screening controls.

**Figure 11:**
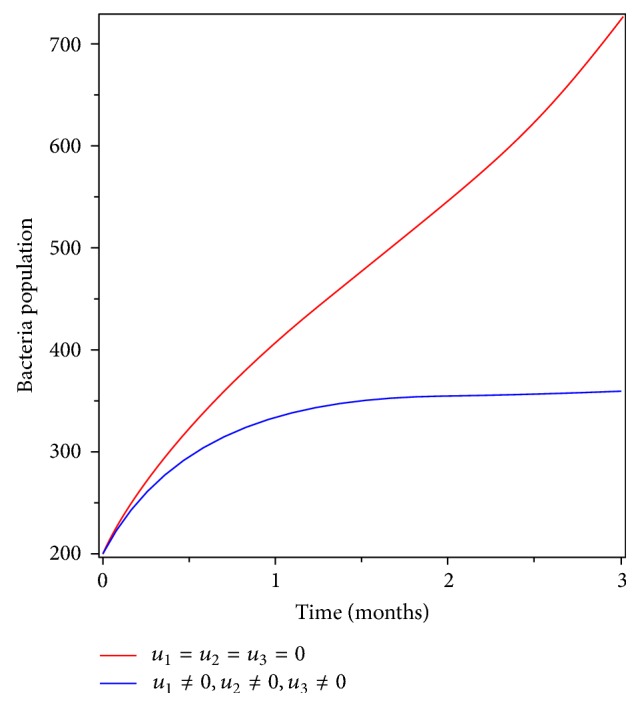
Simulations of the typhoid fever model with prevention, treatment, and screening controls on Salmonella bacteria populations.

In this strategy, we implemented all the three controls (prevention, treatment, and screening) as intervention to eradicate typhoid fever from the community. Figures 10(a) and 10(b) show that the number of infectious individuals and carriers goes to zero at the end of the implementation period. Moreover, Figure 11 shows that the number of Salmonella bacteria population decreased after the implementation of the strategy. Therefore, applying this strategy is effective in eradicating typhoid fever disease form the community in a specified period of time.

## 7. Cost-Effectiveness Analysis

In this section, we identified a strategy which is cost-effective compared to other strategies. To achieve this, we used incremental cost-effectiveness ratio (ICER), which is done dividing the difference of costs between two strategies to the difference of the total number of their infections averted. We estimated the total number of infections averted for each strategy by subtracting total infections with control from without control. To get the total cost of each strategy, we used their respective cost function ((1/2)*w*_1_*u*_1_^2^, (1/2)*w*_2_*u*_2_^2^, and (1/2)*w*_3_*u*_3_^2^) to calculate over the time of intervention. We did not consider strategies that implement one intervention only, due to the reason that one intervention only is not guaranteed to eradicate the disease totally from the community. Those strategies which incorporate more than one intervention are ordered below to be compared pairwise:

  Strategy A (prevention and screening)
  Strategy B (treatment and screening)
  Strategy C (prevention and treatment)
  Strategy D (prevention, treatment, and screening)

We used parameter values in Table 2 to estimate the total cost and total infections averted in Table 3.

First we compared the cost-effectiveness of strategies A and B: ICER(A) = 733.07/11,977 = 0.06, ICER(B) = (733.07 − 800)/(11,977 − 13,805) = 0.037.

This shows that strategy B is cheaper than strategy A by saving 0.037. That means strategy A needs higher money than strategy B. Therefore, we exclude strategy A and continue to compare strategies B and C.

(56) ICERB=80013,805=0.058,ICERC=800−573.1913,805−19,699=−0.039.

Similarly, this comparison indicates that strategy C is cheaper than strategy B by saving 0.039. Therefore, strategy B is rejected and continues to compare strategy C with the last strategy which is D.

(57) ICERC=573.1919,699=0.029,ICERD=573.19−1,104.519,699−19,987=1.845.

Finally, the comparison result reveals that strategy C is cheaper than strategy D by saving 0.029. Therefore, strategy C (combination of prevention and treatment) is the best strategy from all compared strategies due to its cost-effectiveness and healthy benefit.

**Figure 12:**
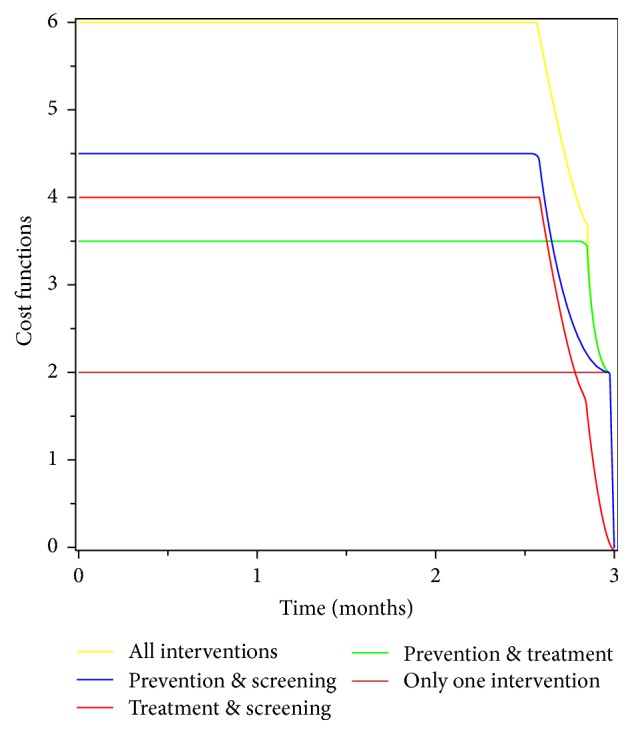
Cost function of the intervention strategies for the period of 3 months.

Moreover, Figure 12 shows that applying only one intervention is cheapest. But we do not consider this because a single intervention is not effective in eradicating the disease. A combination of prevention and treatment strategy is the cheapest of all other combined intervention strategies. The combination of all the three interventions (prevention, treatment, and screening) is the most expensive strategy compared to other strategies.

## 8. Discussions and Conclusions

In this study, a deterministic model for the dynamics of typhoid fever disease is proposed. The qualitative analysis of the model shows that the solution of the model is bounded and positive and also the equilibria points of the model are obtained and their local as well as global stability condition is established. The study also obtained the basic reproduction number and it reveals that for *ℜ*_0_ < 1 there is no possibility of having backward bifurcation. In Section 4, sensitivity analysis of the reproductive number has been carried out. Results from the sensitivity analysis of the reproductive number suggest that an increase in *v*, *K*, *σ*_1_, and *σ*_2_ has the greatest influence on increasing the magnitude of the associated reproductive number which results in the endemicity of typhoid fever.

In Section 5, using Pontryagin's maximum principle, the optimal control problem is formulated and the conditions for optimal control of the disease are analyzed with effective preventive measures (sanitation and proper hygiene controls), treatment regime, and screening. Existence conditions for optimal control are established and the optimality system is developed. Seven intervention strategies are proposed for examining each strategy on the eradication of typhoid. In Section 6, the proposed strategies are investigated numerically and their results are displayed graphically. Cost-effectiveness analysis of the main strategies is done in Section 7, and the results indicate that prevention and the cost put into treatment have a strong impact on the disease control. Effective treatment only without prevention is not the best option in controlling the spread of typhoid fever. Therefore, this finding conclude that adequate control measures which adhered to these control strategies (preventive and treatment) would be a very effective way for fighting the disease and also for cost-effectiveness.

## Supplementary Material

Figure 13 shows control profile . This figure discribes as the control profiles are bounded between 0 and 1.

## Figures and Tables

**Table 1:** Sensitivity indices table.

Parameter symbol	Sensitivity indices
*v*	1
*K*	0.999
*σ*_1_	0.26
*σ*_2_	0.03
*ρ*	0.00506
*μ*	−1.028
*μ*_*b*_	−1
*α*	−0.0592
*θ*	0.009
*β*	−0.00017
*ϕ*	−0.000089

**Table 2:** Parameter values for typhoid fever model.

Parameter symbol	Parameter description	Value	Source
*v*	Salmonella ingestion rate	0.9	Assumed
*K*	Concentration of Salmonella bacteria in foods and water	50000	[14]
*μ*	Human beings natural death rate	0.0247	Assumed
*α*	Typhoid induced death rate	0.052	Estimated
*β*	Treatment rate of infectious diseases	0.002	Estimated
*σ*_1_	Discharge rate of Salmonella from carriers	0.9	Gosh et al., 2006
*σ*_2_	Discharge rate of Salmonella from infective	0.8	Assumed
*δ*	Removal rate from recovered subclass to susceptible subclass	0.000904	Adetunde, 2008
*θ*	Screening rate of carriers	0.2	Assumed
*ϕ*	Removal of carriers by natural immunity	0.0003	Assumed
*ρ*	Probability of susceptible joining carrier state	0.3	Assumed
*μ*_*b*_	Natural/drug induced death rate of bacteria	0.001	Gosh et al., 2006
Λ	Recruitment of human beings	100	Assumed

**Table 3:** Number of infections averted and total cost of each strategy.

Strategies	Description	Total infections averted	Total cost (USD)
A	Prevention and screening	11,977	733.07
B	Treatment and screening	13,805	800
C	Prevention and treatment	19,699	531.19
D	Prevention, treatment, and screening	19,987	1104.5
