# Supplementary figures and images for: Modelling and Optimal Control of Typhoid Fever Disease with Cost-Effective Strategies
Source: Comput Math Methods Med. 2017 Sep 10;2017:2324518. doi: 10.1155/2017/2324518 (PMC5610837; doi:10.1155/2017/2324518)

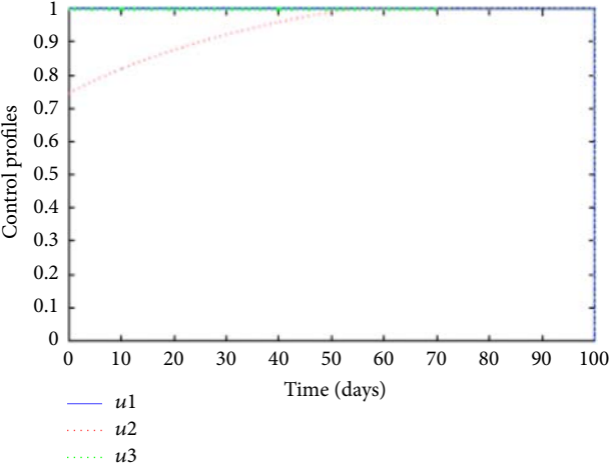

Supplement: Supplementary file 1 — Figure 13 shows control profile . This figure discribes as the control profiles are bounded between 0 and 1. [file 2324518.f1.pdf]
